# Influence of non-thermal plasma on structural and electrical properties of globular and nanostructured conductive polymer polypyrrole in water suspension

**DOI:** 10.1038/s41598-017-15184-0

**Published:** 2017-11-08

**Authors:** Pavel Galář, Josef Khun, Dušan Kopecký, Vladimír Scholtz, Miroslava Trchová, Anna Fučíková, Jana Jirešová, Ladislav Fišer

**Affiliations:** 1 Department of Physics and Measurements, University of Chemistry and Technology, Prague, 166 28 Czech Republic; 2 Institute of Macromolecular Chemistry, Academy of Sciences of the Czech Republic, Prague, 162 06 Czech Republic; 3 Department of Chemical Physics and Optics, Faculty of Mathematics and Physics, Charles University in Prague, 121 16 Prague, Czech Republic

## Abstract

Non-thermal plasma has proved its benefits in medicine, plasma assisted polymerization, food industry and many other fields. Even though, the ability of non-thermal plasma to modify surface properties of various materials is generally known, only limited attention has been given to exploitations of this treatment on conductive polymers. Here, we show study of non-thermal plasma treatment on properties of globular and nanostructured polypyrrole in the distilled water. We observe that plasma presence over the suspension level doesn’t change morphology of the polymer (shape), but significantly influences its elemental composition and physical properties. After 60 min of treatment, the relative concentration of chloride counter ions decreased approximately 3 and 4 times for nanostructured and globular form, respectively and concentration of oxygen increased approximately 3 times for both forms. Simultaneously, conductivity decrease (14 times for globular and 2 times for nanostructured one) and changes in zeta potential characteristics of both samples were observed. The modification evolution was dominated by multi-exponential function with time constants having values approximately 1 and 10 min for both samples. It is expected that these time constants are related to two modification processes connected to direct presence of the spark and to long-lived species generated by the plasma.

## Introduction

Non-thermal (also known as cold or non-equilibrium) plasma (NTP) is an ionized gas created typically by electrical discharges. In contrast to the other types of plasma, NTP possesses following features: (*i*) the generating energy favors electrons and (*ii*) there is only limited transfer of momentum between electrons and heavy particles. Thus, the electrons show high temperature that reaches tens of thousands of Kelvins, while ions temperature is usually near or little above the ambient temperature. Nevertheless, the electron energy distribution is still close to the thermal distribution. These conditions determine the main characteristics and performance of NTP^1,2^. Ionization and chemical processes induced by NTP are related to the electron energy (approximately units of eV). The collisions of these electrons with other particles induce the creation of reactive radicals, charged particles and electromagnetic radiation ranging mainly from ultraviolet to visible spectral region. Therefore, the NTP has potential to initiate various and often unique chemical reactions in a simple, fast, environmentally friendly and cost-effective ways (*e.g*. preparation of low dimensional structures), and promote specific modifications of almost any surface without their thermal damage, including the most resistive materials^3,4^.

This emerging field already showed several diverse applications. One of the most important has been reported in biomedicine and plasma health care^5^. NTP is being used to treat cancer^6^, for sterilization of medical equipment^7^, decontamination and disinfection^8^, as a therapeutic^9^ and drug delivery techniques^10^, for treatment of blood coagulation^11^, to heal wounds and skin diseases^12^, and in cosmetics^13^. Plasma application also proved to be effective tool in polymer science^14,15^ and in development of new organic/inorganic nanostructures and tailoring their functional properties^16^. In particular, plasma assisted polymerization and deposition of thin layers on various surfaces together with nanocrystals formation and intentional modification of their surface properties receive great interest in the scientific community^2^. Based on the recent advances in atmospheric pressure microplasma technology, the NTP also found its application in microelectronics^17,18^ and photovoltaics^19^. The utilization of NTP in environment protection^20^, agriculture^21^ and food industry^22^ is also worth mentioning.

In contrast to above mentioned NTP textile and protective polymers applications, only limited attention has been given to exploitations of NTP related to conductive polymers (CPs). These unique polymers exhibit exceptional electrical and optical properties that in combination with biocompatibility and good mechanical properties lead to a large number of up to date applications ranging from chemical and biological sensors^23^, supercapacitors^24^, smart biomaterial^25^ and photovoltaics^26^ to flexible electronics^27^. It has been shown that NTP technology can provide easy and cost-effective ways of CPs synthesis, especially with regards to specific nanostructured forms^28–30^ and be effective in CPs deposition^31^. However, the advantages of surface termination/functionalization of CPs by NTP, except one pioneering work^32^, remains still unexplored. This approach has potential to effectively deal with tuning of their surface properties and stability which are still challenging topics.

Presented work addresses this issue and reports a complex study of NTP influence on structural, morphological and electrical properties of conductive polymer polypyrrole in globular (PPy-G) and also nanostructured (PPy-NT) form which were dispersed in distilled water. The aim of our research is to determine the effect of NTP exposition on doping level, elemental composition and surface properties of the PPy, which could be afterwards used for intentional tuning of PPy properties. In order to provide the most versatile study, the transient spark plasma technique was applied^33^. The experimental setup for generation of this kind of atmospheric pressure non-thermal plasma is simple, inexpensive and can be used for sample treatment also in liquids that allow generation of wider range of reactive species in comparison with gases^34,35^. Used samples were characterized before and after modification by energy dispersive X-ray spectroscopy (EDX), Fourier transform infrared (FTIR) spectroscopy, Raman spectroscopy and scanning electron microscopy (SEM). Measurement of electrical conductivity of PPy after different modification steps together with its zeta potential (ZP) characterization have been also carried out.

## Results and Discussion

**Figure 1:**
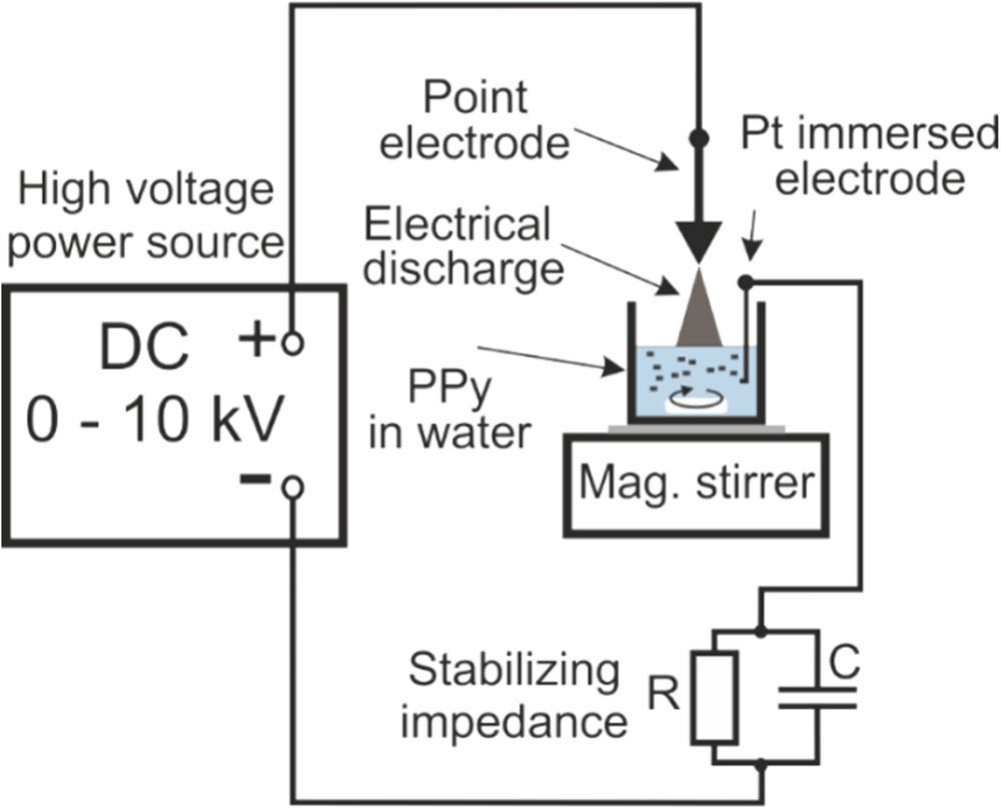
Scheme of non-thermal plasma apparatus. Experimental setup used for the NTP generation and modification of the PPy in distilled water. The discharge in a positive regime of transient spark at atmosphere pressure was used.

The samples of PPy-G and PPy-NT were prepared and treated in water suspension by non-thermal plasma for various time intervals ranging from 1 to 60 min (for experimental setup, see Fig. 1). The results of subsequent sample modifications are as follows.

**Figure 2:**
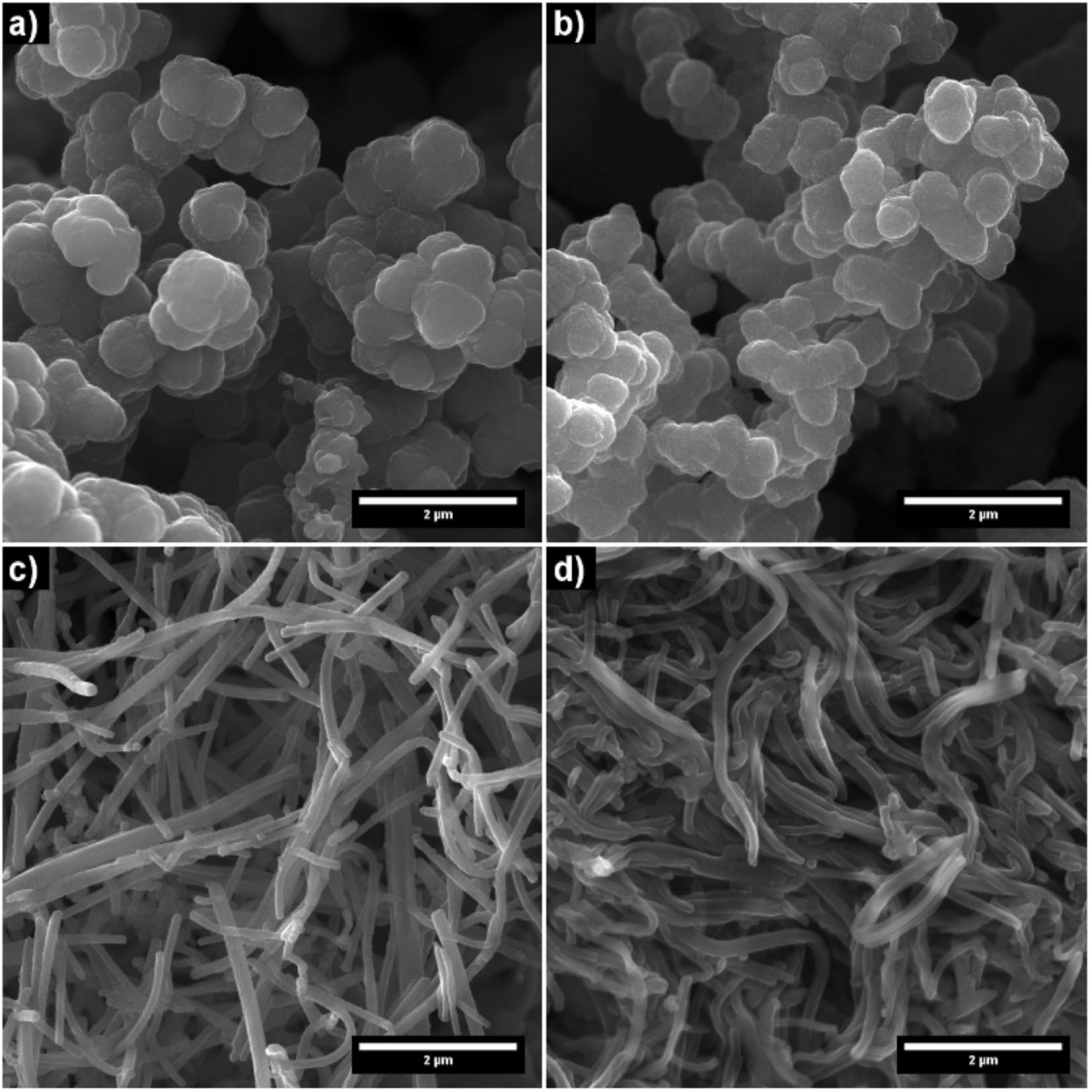
Morphology of PPy observed by scanning electron microscopy. (**a**) as-prepared PPy-G; (**b**) PPy-G after 50 min of plasma treatment; (**c**) as-prepared PPy-NT and (**d**) after 50 min of plasma treatment.

Firstly, morphology of as-prepared and treated PPy samples was characterized. SEM pictures of both PPy-G and PPy-NT without and after 50 min of plasma treatment are presented in Fig. 2. Both polymer structures showed the same shape before and after the treatment. Also, no cross-linking or polymer cutting was observed. Thus, the plasma procedure does not change these morphological properties of both polymer forms that proves previously presented statement of non-destructive nature of NTP treatment^1^.

**Figure 3:**
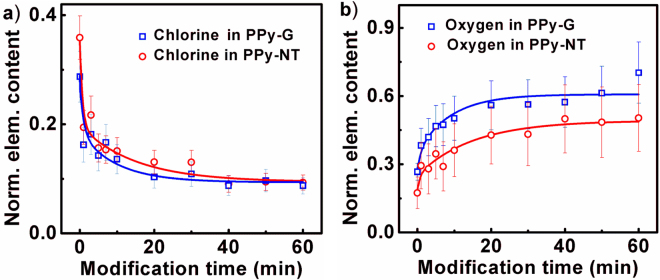
Elemental characterization of the PPy before and after modification using energy dispersive X-ray spectroscopy. Dependence of (**a**) chlorine and (**b**) oxygen content in PPy-G and PPy-NT on time of plasma treatment. Elemental compositions are normalized per pyrrole unit. Solid lines are from the best multiexponential fits of the experimental data. Time constants gained from (**a**) were also applied in approximation of dependences showed in (**b**) (see Table 1).

The dependence of elemental composition (oxygen and chlorine) on the duration of plasma treatment for PPy-G and PPy-NT are shown in Fig. 3. Both elements are normalized per one pyrrole unit excluding elements from remnant molecules MO used in the nanotubes preparation process. Recent work was used for the calculation of oxygen to nitrogen and chlorine to nitrogen ratio, respectively^36^. Chlorine is incorporated into both PPy-G and PPy-NT as chloride counter anions (so called dopant) and its content has essential influence on electrical conductivity of PPy. As can be seen in the Fig. 3a, the relative content of chlorine decreased with increasing exposition time from 0.28 and 0.37 to 0.087 and 0.095 for PPy-G and PPy-NT, respectively. Under used experimental conditions, the deprotonation of PPy is most probably carrier out though creation of hydrochloric acid^37^. The modifications time evolutions can be approximated well by two exponential function (Table 1) using time constants *τ*_1_ and *τ*_2_ having values of 0.7 and 13 min, and 1.1 and 9 min for PPy-G and PPy-NT, respectively. Therefore, two processes should be responsible for the polymer modifications. Their origin can be related to the direct presence of discharge, and also to short and long-lived species plasma generated from the surrounding substances. The exact mechanism of these processes will be given (*vide infra*). The ratio between amplitudes of exponential functions *A*_1_ and *A*_2_ is 1.7 and 1.9 for PPy-G and PPy-NT, respectively. Higher influence of the first modification process is in good agreement with the large initial drop of chlorine in both samples.

**Table 1:** Fitting parameters of chlorine and oxygen concentration evolution in PPy during plasma exposure.

	PPy-G			PPy-NT		
	*τ*_1_(min)	*τ*_2_(min)	*A*_1_/*A*_2_	*τ*_1_(min)	*τ*_2_(min)	*A*_1_/*A*_2_
Chlorine	0.7	13	1.7	1.1	9	1.9
Oxygen	0.7	13	0.28	1.1	9	0.16

Values of the time constants *τ*_1_ and *τ*_2_, and ratio of corresponding amplitudes *A*_1_/*A*_2_ of the multiexponential functions used to approximate the evolution of PPy normalized elemental composition during plasma treatment. The time constants gain from chlorine evolution fit were applied also in the case of oxygen. Error of time constants were about 30% of the value.

In contrast to the chlorine, oxygen presence is not native in both PPy samples before the NTP treatment. Nevertheless, it is apparent from the Fig. 3b that content of oxygen in both PPy-G and PPy-NT steeply increases during the treatment from 0.25 and 0.15 to 0.68 and 0.50 for PPy-G and PPy-NT, respectively. To prove the assumption of presence of two modification processes, these evolutions were successfully approximated by the same time constants as in the case of chlorine. In this case, the dominating process seems to the one characterized by *τ*_2_ that is demonstrated by the ratio of *A*_1_/*A*_2_ having values of 0.28 and 0.16 for PPy-G and PPy-NT, respectively (Table 1). Excluding the oxygen molecules of MO counter anions, observed content of oxygen may originate from: (*i*) air oxygen adsorbed in form of O_2_^−^ anions, (*ii*) -C=O carbonyl groups of *α* and *β* carbons of pyrrole ring, (*iii*) -C=O carbonyl or –COOH carboxyl groups originating from pyrrole ring opening and (*iv*) finally from -OH hydroxyl group resulting from interaction of PPy chain with moisture or oxygen. As these EDX results are unspecific, FTIR and Raman spectra are important to evaluate more detailed information^37^.

### Fourier-transform infrared and Raman spectra

**Figure 4:**
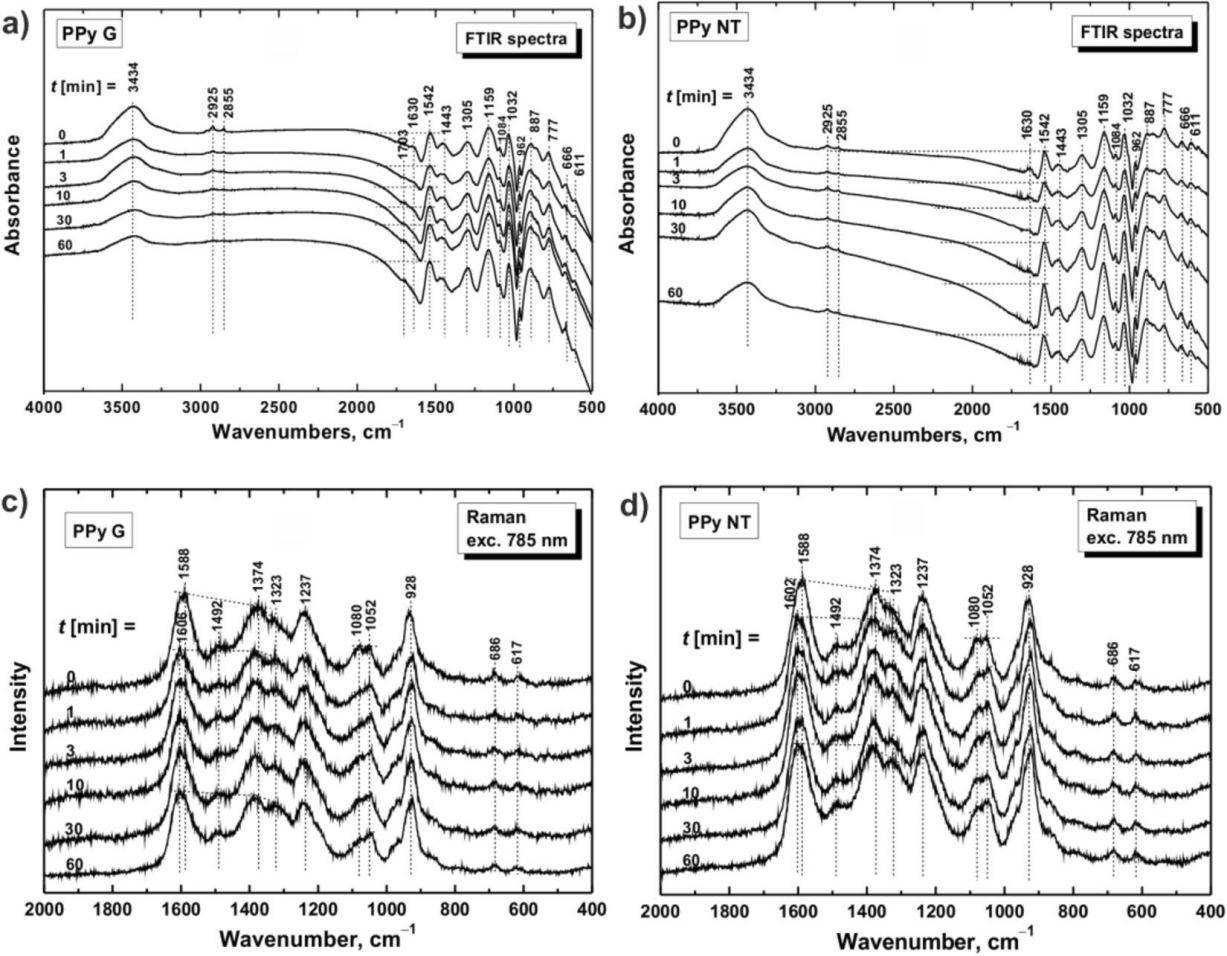
Fourier-transform infrared and Raman spectra of PPy before and after modification. FTIR spectra of (**a**) PPy-G and (**b**) PPy-NT and Raman spectra of (**c**) PPy-G and (**d**) PPy-NT treated with plasma during various time periods (*t*): 1, 3, 10, 30, and 60 min. Raman spectra were measured upon excitation at 785 nm.

Infrared spectrum of the initial PPy-G (Fig. 4a) shows the main bands of PPy which were described in detail elsewhere^38–40^. These bands are clearly observed also in the spectra of modified samples from which we conclude that the molecular structure of PPy is preserved during the plasma treating (Fig. 4a). However, infrared spectra of PPy-G treated with plasma during various time consecutively change with increasing of the exposition time. The bands of impurities related to water presence (3440 and 1630 cm^−1^) and the aliphatic impurities bands (2925 and 2855 cm^−1^) consecutively decrease. It may be related to the better dispersion of the samples in potassium bromide (FTIR pellet) due to the breaking of hydrogen bonding which leads to their stone-like structure. We observe decrease of the absorption in the interval above 1600 cm^−1^. Practically no band shifts occur. Observed changes correspond to the deprotonation of the samples after plasma treating^37^.

Infrared spectrum of original PPy-NT contains the main bands of the globular PPy, so the molecular structure of polymer chains is similar for both morphologies (Fig. 4b). Only very small traces of MO are detected in the spectra of PPy-NT. Samples were difficult to disperse in potassium bromide due to their stone-like structure. It is reflected in more profound bands of humidity and of aliphatic impurities of the potassium bromide pellets. After plasma treatment, the infrared spectrum of the original PPy-NT (Fig. 4b) changes. Similar to the PPy-G, we detected decrease of the absorption in the region above 1600 cm^−1^ and of water related impurities, but no change in aliphatic impurities bands. The molecular structure of PPy-NT is also being preserved during plasma treatment^38–40^. The band of C–C stretching vibrations in the pyrrole ring (1542 cm^−1^) is shifted to higher wavenumbers as well as the band of C–N stretching vibration in the pyrrole rings at 1455 cm^−1^. We have also observed shift of signal from breathing vibrations of the pyrrole rings (band at 1159 cm^−1^) and the N–H^+^ deformation vibrations (peaks at 1032 and 1092 cm^−1^) to higher wavenumbers during the plasma treatment. All these changes were observed also during deprotonation of PPy with alkaline medium^37^. These changes are more profound in the spectra of nanotubular PPy than in case of granular PPy. We suppose that interaction with MO is partly disrupted by plasma treating.

The existence of polarons and bipolarons in the protonated PPy can be determined by application of Raman spectroscopy technique using excitation at 785 nm^37^. In the spectrum of PPy-G (Fig. 4c) we detect signal related to the C=C in-ring of C–C inter-ring vibrations and the stretching vibrations in cation of the PPy backbone (1588 cm^−1^)^41,42^. The maximum of the latter band shifted to 1606 cm^−1^ after plasma treatment of the samples. It can be referred to the C=C backbone stretching vibrations in short conjugation length of deprotonated PPy^41^. The double peak at about 1374 and 1323 cm^−1^ is associated with the ring-stretching vibrations of pyrrole. The latter increased after plasma treatment which corresponds to the deprotonation^42^. The deprotonation of PPy-G during plasma treatment is also supported by the intensity increase of the lower wavenumber part of the double peak situated at 1080 and 1052 cm^−1^)^41^.

The deprotonation of the PPy-NT after treatment with plasma is also detected in the Raman spectra (Fig. 4d). The maximum of the band of PPy backbone observed at 1588 cm^−1^ in the original PPy-NT spectrum shifted to higher wavenumbers^41,42^. Also in the case of PPy-NT, the plasma treatment caused relative intensity increase of the second maximum of double peak at 1323 cm^−1^ ^41^. This peak has been connected with less conducting PPy^43^. The relative intensity in the double peak localized at 1080 and 1052 cm^−1^ also changed, the latter maximum which corresponds to the vibrations in neutral units of polypyrrole base relatively increased^41^. Based on the Raman spectra the deprotonation of PPy-NT is less pronounced than it is in case of PPy-G^37^.

### Electrical conductivity and Zeta potential

**Figure 5:**
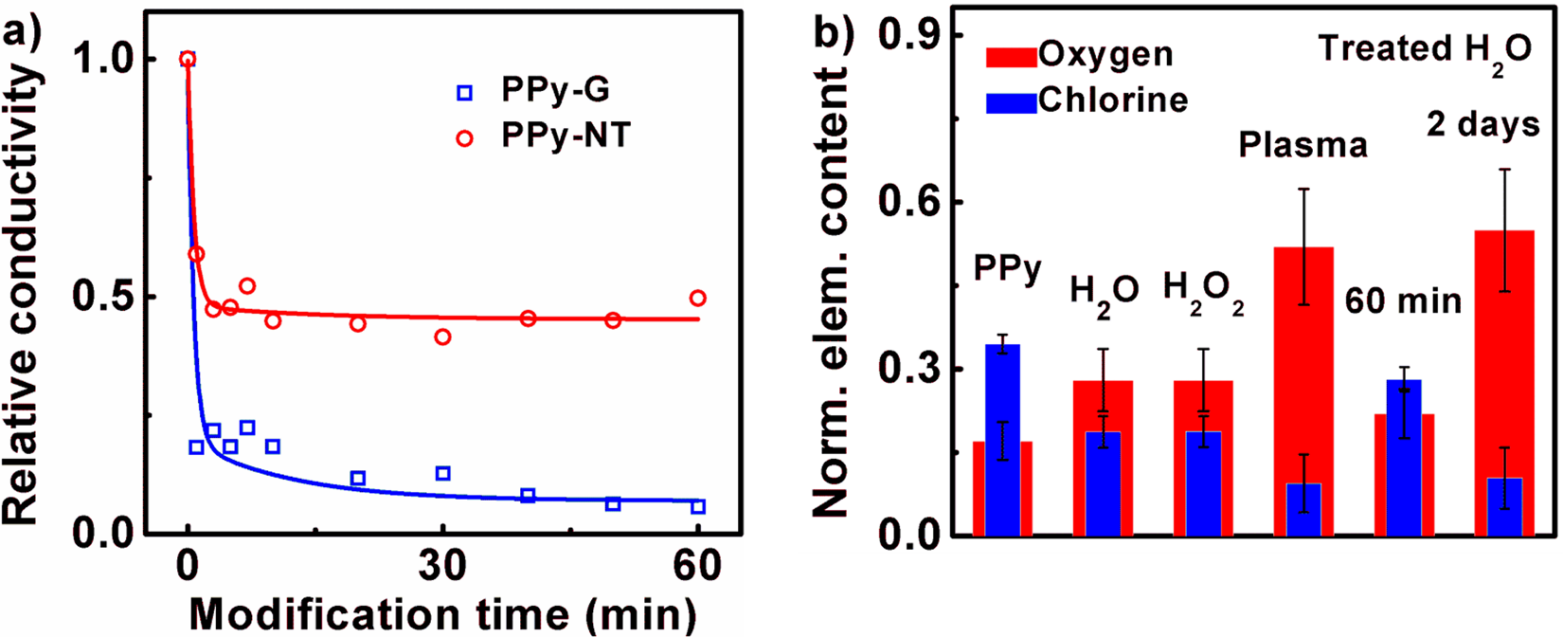
Evolution of PPy electrical conductivity before and after modification, and elemental composition of PPy using various solvents. (**a**) Dependence of PPy-G and PPy-NT relative electrical conductivity on time of plasma treatment. Solid lines are from the biexponential fits obtained using the same time constants as in Fig. 3 and Table 1 (PPy-NT *τ*_1_ = 1.1 min and *τ*_2_ = 9 min, PPy-G *τ*_1_ = 0.7 min and *τ*_2_ = 13 min). Amplitude ratio *A*_1_/*A*_2_ was 5.8 (PPy-G) and 18.1 (PPy-NT). Initial values of electrical conductivity of PPy-G and PPy-NT measured upon pressing weight of 5 kg were 0.78 and 2.1 S/cm, respectively. Passing current of discharge during treatment was 0.9 mA. (**b**) Comparison of chlorine and oxygen normalized elemental content of neat PPy-NT, after 2-day treatment by distilled water and peroxide, after 60 min direct plasma treatment in distilled water, and after 60 min and 2-day treatment in PAW.

The dependences of conductivity on plasma treatment duration applied on both PPy-G and PPy-NT in water suspension are shown in Fig. 5a. The conductivity strongly decreased during the first minutes of treatment regardless the PPy structure. The course of both PPy-G and PPy-NT electrical conductivity dependencies is in good agreement with EDX, FTIR and Raman characteristic and similarly as in the case of EDX measurements can be approximated well by two exponential function using the same time constants (Table 1). High sensitivity of the conductivity to the first modification process is demonstrated by the amplitude ration between *A*_1_ and *A*_2_ having values 5.8 and 18.1 for PPy-G and PPy-NT, respectively. Thus, the loose of dopants by deprotonation and hence decrease of polaron amount is, according to our opinion, main reason for decrease of electrical conductivity in both PPy morphologies. In addition, taking into account that after the 1 hour NTP treatment the conductivity of PPy-NT and PPy-G decreased about 54% and 93%, respectively, while the resulting chlorine concentrations of both samples were comparable (Fig. 3), the change of the conductivity should be also dependent on the oxygen presence. The NTP creates several sources of oxygen in distilled water during discharge, among the most important are ozone (O_3_), hydrogen peroxide (H_2_O_2_) and oxygen radicals (*vide infra*). According to the Figs 3 and 4, PPy-NT is more durable from the point of oxygen absorption in comparison with PPy-G. This can be caused, surprisingly, by more compact macroscopic structure of PPy-NT which reminiscent sand particles prone to absorption of distilled water containing sources of oxygen. Similar behavior was previously reported on PPy doped by aromatic sulfonic acids^44^. Planar backbone of aromatic dopants create more oriented backbone of PPy and hence stiffed material (from macroscopic point of view). Moreover, small circular dopants like chloride counter anions create feather like material dispersible into small particles. Therefore, oxygen in various forms penetrate worse into PPy-NT in comparison with PPy-G. PPy-NT also resist to nucleophilic attack of oxygen resulting in creation of carbonyl group on pyrrole ring, as can be seen from FTIR spectra of PPy-G at 1703 cm^−1^. Creation of carbonyl group in PPy-G can be observed on dependency of electrical conductivity on exposition time as slow but continual decrease of electrical conductivity. Therefore, the overall evolution of PPy-G electrical conductivity is driven by both deprotonation and oxygen degradation. The higher stability of PPy-NT toward oxygen attack in comparison with PPy-G is gratifying from the application perspectives.

Influence of plasma treatment on PPy surface properties was also studied by Zeta-potential measurements. As can be seen in Table 2, the presence of non-thermal plasma significantly changed characteristics of both PPy types. In case of PPy-G, the intensity of original peak centered approximately −30 mV started to decrease and shifted to higher values while a new peak was observed (about −10 mV) during the initial phase of modification. The original peak banished after 10 minutes of modification and no further changes of zeta-potential characteristics of PPy-G was detected with additional NTP modification (Figure S3 in *Supporting Information*). In contrast to the PPy-G, the ZP of PPy-NT was getting more negative with increasing modification time. Also in this case, we observed almost immediate reaction of PPy surface properties on plasma presence, but the following change of peak position proceeded continuously with increasing NTP modification time (Figure S3 in *Supporting Information*). Similarly, no changes of ZP peak centered approximately −60 mV are observed after 10 min of modification. These phenomena are in good agreement with the presence of two modification processes. According to the duration of the changes, they should be related mainly to the increase of oxygen concentration (Fig. 3b). This statement is also consistent with the discrepancy in courses of the modifications for both samples. Different oxygen related processes for both PPy types were already discussed. The resulting values of ZP for PPy-G (−10 mV) and PPy-NT (−60 mV) can by interpreted in the way of their stability in water. Meanwhile the PPy-G nanoparticle should agglomerate in time, the PPy-NT showed high repulsion and therefore, good stability in water. Contrast between this observation and results gained by Fourier-transformed infrared spectroscopy can be caused by using different solutants. During the measurement of the PPy-NT (all modification times) and for PPy-G (0 min) we observed strong charging and high conductivity of measured samples, therefore, they have been measured by alternative approach, as mentioned in the Experimental part of the article. High conductivity of PPy in these states, were proved by conductivity measurements (Fig. 5a).

**Table 2:** Zeta-potential characterization of PPy before and after plasma treatment.

Treat. time	PPy-G			PPy-NT
	Position ± FWHM of ZP peaks (mV)			Position ± FWHM of ZP peak (mV)
**0**	−29 ± 11			−12 ± 5
**1**	−12 ± 6		−44 ± 7	−23 ± 7
**3**	−8 ± 7		−35 ± 9	−23 ± 6
**10**	−18 ± 10			−61 ± 10
**30**	−15 ± 10			−63 ± 14
**60**	−14 ± 10			−59 ± 14

Dependence of PPy-G and PPy-NT zeta potential (ZP) bands maxima and full width half maximum (FWHM) on plasma modification time.

### Origin of NTP modification processes

To elucidate the origin of PPy modification processes the following comparative study have been carried out. We have characterized elemental content of chlorine and oxygen in neat PPy-NT and after 60 min of plasma treatment in water suspension, after letting the neat PPy-NT for 2 days in distilled water and peroxide, and after 60 min and 2 days in the plasma activated water (PAW), see Fig. 5b. PAW (also known as water of dead) is distilled water previously treated by the NTP before adding the polymer. It was proved that PAW is effective in disinfection and other health related processes^45,46^. As can be seen, not only NTP can cause concentration changes of monitored elements. The polymer after 2 days in distilled water also showed increase of oxygen concentration (from 0.15 to 0.28) and decrease of chlorine (from 0.37 to 0.19), but these changes were lower than after 1 min of plasma treatment. Surprisingly, similar changes were observed also using the hydrogen peroxide that is generally taken as a main modification factor in PAW. Even weaker changes of elemental content were detected using the modified distilled water for 60 min (0.28 and 0.22 for chlorine and oxygen, respectively). However, after two days the modification caused by PAW reached state comparable to the direct NTP treatment. Based on these results, it seems that the first fast modification process (approximately 1 min) is related to the presence of NTP. It can be caused by the creation of short-lived particles that are present in the active plasma region only (i.e. atomic oxygen O, singlet oxygen ^1^O_2_, superoxide anion O_2_^−^, atomic nitrogen N, excited nitrogen N_2_(A), nitric oxide NO·, OH^−^ anion, or OH· radical)^47^. The second process can be interpreted as reaction with long-lived plasma generated species in the suspension, except hydrogen peroxide that was proved to be not more effective than standard distilled water. The presence of long-lived particles may be generated e.g. by following reactions from short-lived particles detected by emission spectra (see Figure S1)^48^:

$$\begin{array}{c}{\rm{O}}+{{\rm{O}}}_{2}+{\rm{M}}\to {{\rm{O}}}_{3}+{\rm{M}}({{\rm{N}}}_{2},{{\rm{O}}}_{2})\\ {{\rm{N}}}^{\ast }+{{\rm{O}}}_{2}\to {\rm{NO}}+{\rm{O}}\\ {\rm{NO}}+{{\rm{O}}}_{3}\to {{\rm{NO}}}_{2}+{{\rm{O}}}_{2}\\ {\rm{O}}+{{\rm{NO}}}_{2}+{\rm{M}}\to {{\rm{NO}}}_{3}+{\rm{M}}\\ {{\rm{NO}}}_{2}+{{\rm{NO}}}_{3}+{\rm{M}}\to {{\rm{N}}}_{2}{{\rm{O}}}_{5}+{\rm{M}},\end{array}$$

where M denotes the third particle or molecule that carries off the excess energy and N^*^ denotes the electronically excited state of nitrogen. The list of relevant long-lived species can contain up to tens of different members (e.g. ozone O_3_, NO_3_^−^, NO_2_^−^) and its role in the longer modification process can also play changes of the suspension physical properties like pH. For that reason, it is logical to expect that the second process is a result of more effects. This statement is in agreement with the time evolutions of pH, and NO_2_^−^ and NO_3_^−^ concentrations in the suspension during the plasma treatment (Figure S4). The changes of pH and NO_3_^−^ showed, in accordance with the second modification process (characterized by *τ*_2_), also exponential behavior, however, values of the related time constants were much longer then *τ*_2_ (both approximately 20 min, Figure S4a,c). Thus, more effects (species) need to be taken into account in the interpretation of the *τ*_2_ origin, specially some showing faster temporal changes (see NO_2_^−^ time evolution, Figure S4b). Nevertheless, the deciphering of the exact *τ*_2_ origin is out of the scope of this report.

The essential observation is also the comparable modification of treated water after 2 days and NTP presence for few tens of min. Although, the presence of NTP can cause observed changes much faster than treated water, it is not necessary for their creation that extend application range of above described technique.

We are aware that for full exploitation of NTP modifications of conductive polymer more work needs to be done. Mainly, the research using different solvents and protective atmosphere should be carried out in order to obtain various modification of the polymer. However, the aim of this paper is the proof of principle that NTP can effectively, fast, non-destructively and cost-effectively modify conductive polymers.

## Conclusion

In summary, influence of NTP presence on morphological, structural and physical properties of globular and nanostructured conductive polymer polypyrrole in distilled water were characterized using SEM, EDX, FTIR, Raman spectroscopy and measurements of conductivity and zeta-potential. Effective termination modification of both samples (oxygen increase approximately 3 times) and deprotonation (~3 and 4 times for PPy-G and PPy-NT, respectively) were proved while no morphological changes were observed. These changes were related to decrease of conductivity of both samples (14 and 2 times for PPy-G and PPy-NT) and zeta-potential. The modifications were dominated by two processes characterized by time constants *τ*_1_ and *τ*_2_ of values 0.7 and 13, and 1.1 and 9 min for PPy-G and PPy-NT, respectively. The origin of the processes should be related to the presence of NTP and/or short-lived species (*τ*_1_) and long-lived species (*τ*_2_). The differences in obtained results for both polymer forms were interpreted based on compactness of their structure and presence of residual structure guiding agent in the nanostructured form. We also proved that comparable modifications can be realized only by NTP treated distilled water, however longer time period is needed (approximately 2 days). Reported results can be used as a basis for more complex and precise modifications of conductive polymers by NTP using different solutions and protective atmosphere (*i.e*. termination by specific functional groups, protonation/deprotonation, tuning of zeta-potential and intentional aging).

## Methods

### Synthesis of globular and nanostructured polypyrrole

Pyrrole (Sigma-Aldrich, Germany), iron(III) chloride hexahydrate (Sigma-Aldrich, Germany), methyl orange *i.e*. (4-[4-(Dimethylamino)phenylazo]benzenesulfonic acid sodium salt (Fluka, Switzerland), ethanol (Penta, Czech Republic), acetone (Penta, Czech Republic) were used as received. Distilled water was used in all reactions. PPy-G was synthesized by dissolution of 2 ml pyrrole monomer (0.03 mol) in 600 ml of distilled water which was cooled by thermostat to 5 °C and subsequent dropwise addition of 8.12 g iron(III) chloride hexahydrate (0.03 mol) dissolved in 69 ml of distilled water^49^. Thus, the concentration of pyrrole and iron(III) chloride hexahydrate in reaction solution was 45 mM. Molar ratio of both reactants – pyrrole: iron(III) chloride hexahydrate – was 1:1. The reaction solution was stirred for 24 h. Finally, the black precipitate of PPy was filtered out and washed by ethanol and distilled water. Filtered black gel was dried in vacuum at 40 °C for 1 week and then grinded by mortar and pestle into the fine black powder.

PPy-NT was prepared by well-developed technique published in paper^49^ using structure guiding agent methyl orange (MO). 2 ml of pyrrole monomer (0.03 mol) was dissolved in 600 ml of 2.5 mM solution of methyl orange. Solution was cooled to 5 °C and stirred at constant speed. 8.12 g of iron(III) chloride hexahydrate (0.03 mol) was dissolved in 69 ml of distilled water and then added dropwise into the reaction solution. After 24 hours, the black precipitate was filtered and cleaned by Soxhlet extraction using acetone for 1 week. Black gel of PPy nanotubes was dried in vacuum at 40 °C for 1 week and then grinded by mortar and pestle into the fine black powder.

### Non-thermal plasma treatment

Detailed description of the experimental setup can be found elsewhere^45,50^. Briefly, the NTP was generated by non-commercial experimental open air apparatus, using the electrical discharge burning between the point electrode, represented by the tip of a standard syringe needle (Medoject (0.6 × 25) mm), and plane electrode, realized by suspension surface (Fig. 1). The suspension surface was electrically connected with the negative pole of power source (DC high voltage source HT 2103, Utes, Brno) by an immersed glass sealed platinum wire via stabilizing ballast impedance comprising parallel connection of 10 MΩ resistor and capacitor of 250 pF. NTP was used to modify both the PPy-G and PPy-NT (8 mg) suspended in distilled water (2 ml). The plasma treatment was carried out for various time intervals ranging from 1 to 60 min. The source voltage of 7.5 kV and average current of 0.9 mA were kept in all cases. Emission spectrum of applied discharge is reported in *Supplementary Information* on Figure S1. The magnitude of current was controlled by the distance (about 3.5 mm) between the tip of point electrode and water surface. The used electrical discharge burned in a regime of positive transient spark (TS) what is a DC-driven discharge with periodic forming of streamers transforming to a very short spark pulse of peak current of several amperes. Despite of high current peak the temperature of generated plasma remains low and it cannot achieve the conditions of local thermodynamic equilibrium^33^. In our configuration, the repetition frequency of TS was 3.3 ± 0.1 kHz, the duration of spark (the current peak) was 80 ± 10 ns, the maximal current in each peak was approximately of 9 A.

To obtain sufficient amount of sample for all scheduled characterization procedures, all exposures were repeated two and four times for both PPy-G and PPy-NT, respectively. Reproducibility of PPy modification was studied using the same exposition time interval resulting in about 1% standard deviation of N and Cl elemental composition (verified by EDX measurements). More details about reproducibility measurements and deviation calculation are presented in *Supplementary Information* (Equation S1 and Figure S2). After the NTP treatment, the suspension was filtered through filtering paper, rinsed by 30 ml distilled water and dried in vacuum at 40 °C for 3 days. Finally, all doses of modified PPy for the same time periods were poured together and grinded by mortar and pestle. Closer description of the characterization methods used in this research is given in *Supplementary information*.

### Energy dispersive X-ray spectroscopy

MIRA 3 LMH (Tescan, Czech Republic) using 10 kV of accelerating voltage was employed for SEM measurement. Magnification was 30 000×. Energy dispersive X-ray spectroscopy (EDX) EDX measurement were performed by Bruker Quantax 200 with 6|10 XFlash detector using 15 kV of accelerating voltage.

### Fourier-transform infrared spectroscopy

Fourier-transform infrared spectra of the powders dispersed in potassium bromide pellets were registered using a Thermo Nicolet NEXUS 870 FTIR Spectrometer with a DTGS TEC detector in the 400–4000 cm^−1^ wavenumber region.

### Raman spectroscopy

Raman spectroscopy experiments were carried out on a Renishaw InVia Reflex Raman microspectrometer. As a source of excitation light were used NIR laser diode generating continuous laser beam at 785 nm. To focus the excitation beam on the sample mounted on 2D motorized stage were used a research-grade Leica DM LM microscope with ×50 magnification objective. The scattered light was analyzed with a spectrograph using a holographic grating 1200 lines mm^−1^. As a light detector was used Peltier-cooled CCD detector (576 × 384 pixels).

### Measurement of electric conductivity

The electrical conductivity of PPy samples were obtained using noncommercial setup for powder characterization. Construction of the setup was previously reported^51^. Measurement were made in a special insulating chamber with a glass column equipped with four-probe electrodes. This chamber allows measurement of powders (sample weight around 200 mg) under defined contact pressure (up to 4 MPa). The glass column is filled by a measured sample; both ends are sealed by electrodes, height of the sample inside the column is measured by a micrometer. The contact pressure is measured by balances and calculated from a downforce and geometrical dimensions of the sample. HP 34401 ammeter and voltmeter are used for measurement of electrical current and voltage passing through the sample respectively. HP E3631A serves as source of electrical current. Values of specific conductivity are evaluated from Ohm’s law and known geometrical dimensions.

### Zeta-potential characterization

PPy zeta-potential (electrokinetic potential) measurements were carried out using Zetasizer Nano ZS (Malvern) device. The samples were illuminated by the 633 nm line of a He-Ne laser and signal was detected in a backscattering geometry. The zeta-potential values of PPy were calculated by Zetasizer software in general purpose (normal) resolution mode. Generally small amount of PPy was dispersed in deionized water in order to obtain optically clear solution and correct measurement. Since some samples showed significant conductivity, the measurement was modified by addition of clean deionized water to PPy solution until the lowest measurable concentration was obtained, in order to prevent interaction between individual PPy nanoparticles and to minimize their surface charging by external measuring current.

### Availability of data and materials

All relevant data are published within the paper and its supporting additional files.

## Electronic supplementary material

Supplementary Information
