# Supplementary material for: Influence of non-thermal plasma on structural and electrical properties of globular and nanostructured conductive polymer polypyrrole in water suspension
Source: Sci Rep. 2017 Nov 8;7:15068. doi: 10.1038/s41598-017-15184-0 (PMC5678096; doi:10.1038/s41598-017-15184-0)
Supplement: Supplementary file 1 — Supplementary Information [file 41598_2017_15184_MOESM1_ESM.pdf]

Supplementary Information

Influence of non-thermal plasma on structural and electrical properties of globular and nanostructured conductive polymer polypyrrole in water suspension

Pavel Galář[1*], Josef Khun[1], Dušan Kopecký[1], Vladimír Scholtz[1], Miroslava Trchová[2], Anna Fučíková[3], Jana Jirešová[1] and Ladislav Fišer[1].

[1] Department of Physics and Measurements, University of Chemistry and Technology, Prague, 166 28 Prague 6, Czech Republic

[2] Institute of Macromolecular Chemistry, Academy of Sciences of the Czech Republic, 162 06 Prague 6, Czech Republic

[3] Department of Chemical Physics and Optics, Faculty of Mathematics and Physics, Charles University in Prague, 121 16 Prague 2, Ke Karlovu 5, Czech Republic,

[*]*corresponding author: P. Galář, phone: +420 222 443 307, fax: +420 220 444 334, email: pavel.galar*@vscht.cz

Calculation of deviation. To characterize the statistical error of our modification procedure the standard deviation of mean was calculated for six sequential 3 min-long modifications using the following formula:

$$\bar{S} = a\sqrt{\frac{\sum_{i=1}^{N}(\bar{X}_i - X)^2}{N(N-1)}}, \qquad (S1)$$

where the $\bar{S}$ is standard deviation of the mean, N is total number of measurements, $\bar{X}$ is mean of measured parameter, X_i is value of each measured parameter and a is a correction parameter related to number of repetition.

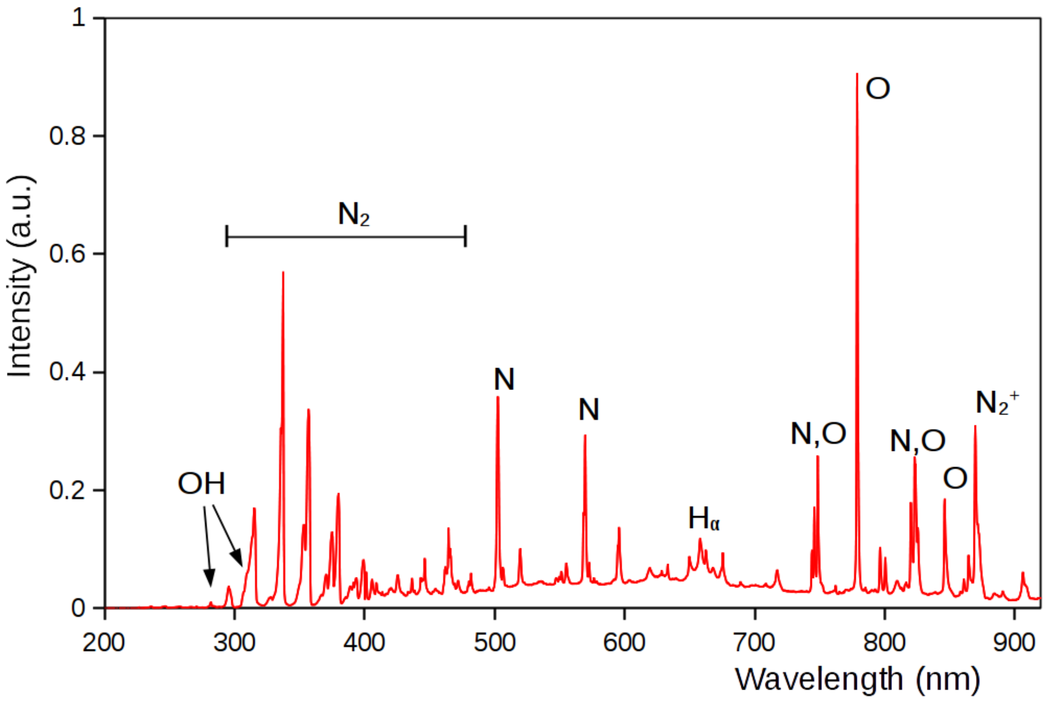

Figure S1. Emission spectra discharge in a positive regime of transient spark at atmosphere pressure used for the NTP generation. Passing current of the discharge was 0.9 mA.[1]

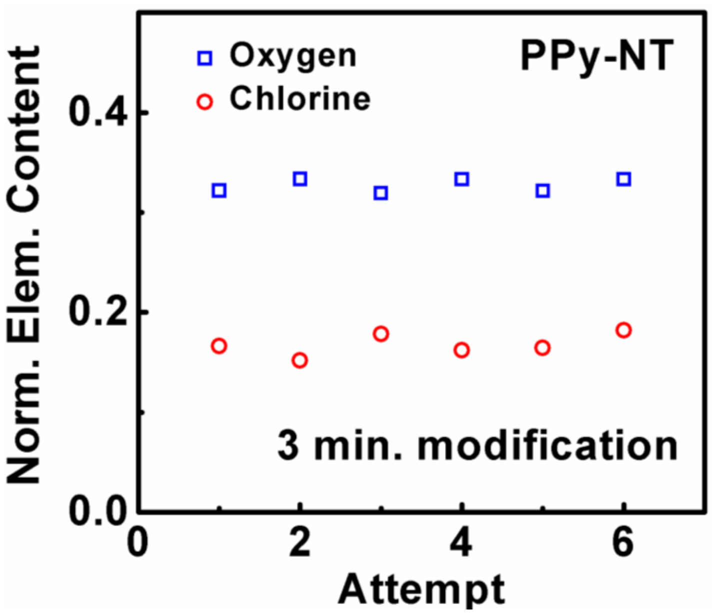

Figure S2. Dependence of oxygen (blue squares) and chlorine (red circles) normalized elemental concentration in PPy-NT after 6 repetitions of 3 min-long plasma treatment. Elemental compositions of both atoms are normalized per pyrrole unit.

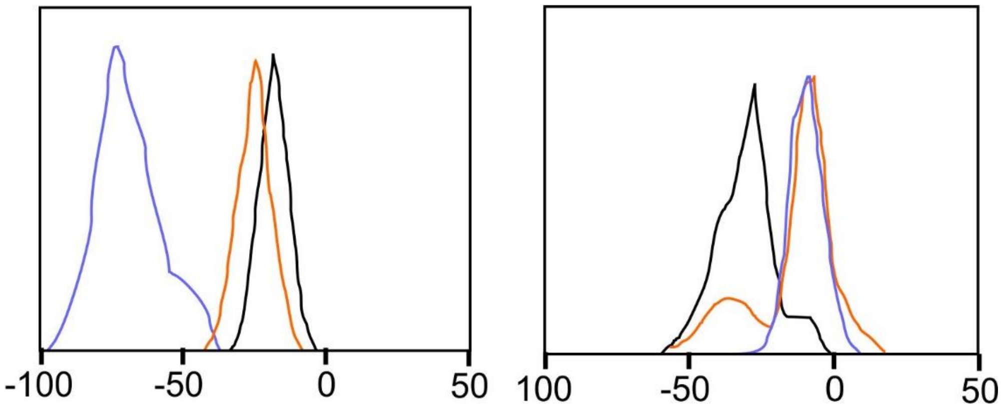

Figure S3. Individual zeta-potential curves for PPy-NT (left) and PPy-G (right) treated by NTP for various time periods (black 0 min, orange 3 min, blue 60 min).

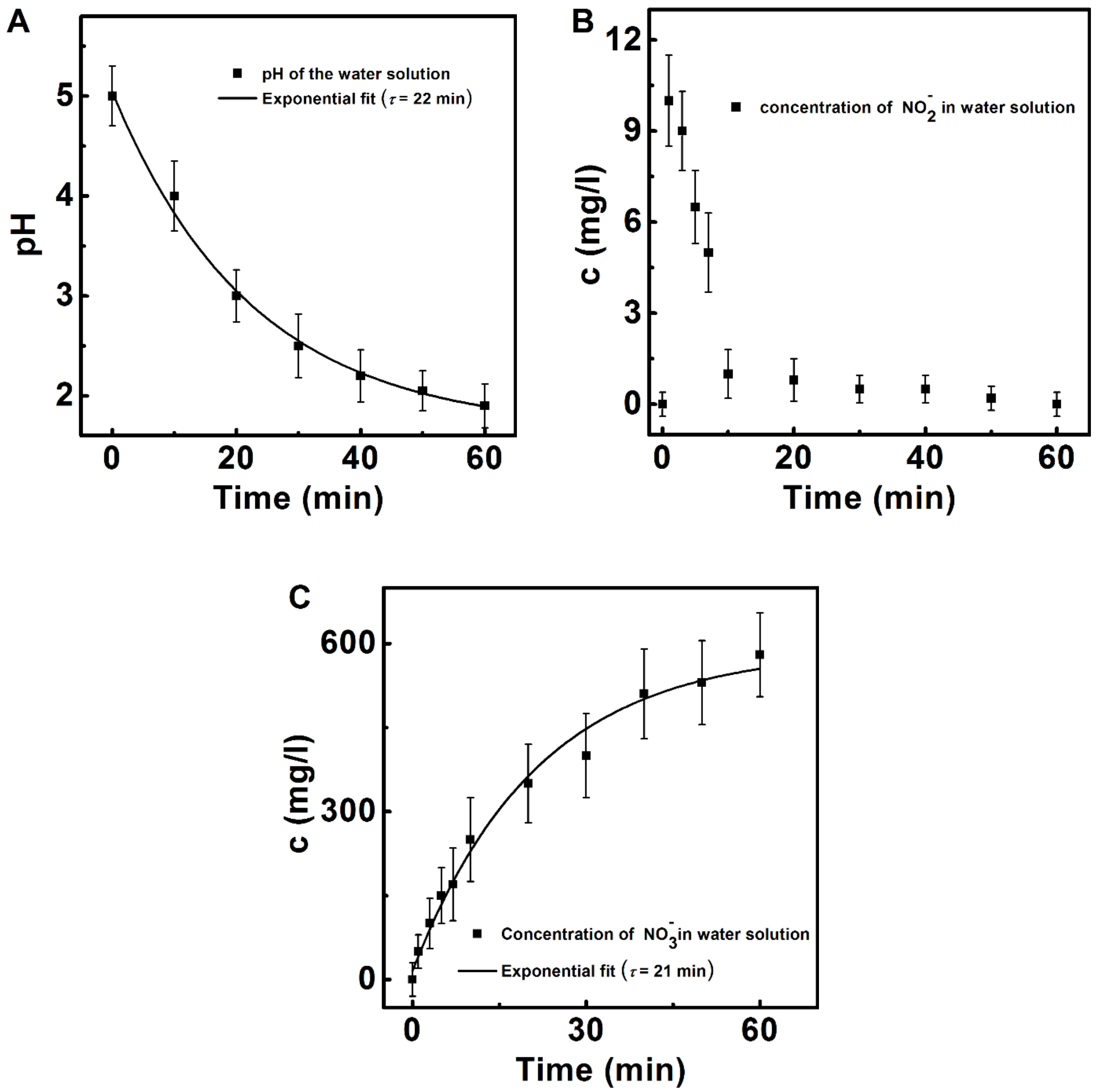

Figure S4. Evolution of (a) pH and concentration of (b) NO_2^- and (c) NO_3^- in the distilled water suspension with PPy-NT during the plasma treatment. Passing current of discharge during treatment was 0.9 mA. Error bars were calculated using standard deviation of mean. Dependences presented in (a) and (c) were approximated by the exponential fit showing time constants τ = 22 and 21 min, respectively (solid lines).

1 Kaushik, N. Uddin, N., Sim, G. B., Hong, Y. J., *et al.* Responses of Solid Tumor Cells in DMEM to Reactive Oxygen Species Generated by Non-Thermal Plasma and Chemically Induced ROS Systems. *Sci. Rep.* **5**, 8587; DOI: 10.1038/srep08587 (2015).
